# Supplementary material for: Accelerated viral dynamics in bat cell lines, with implications for zoonotic emergence
Source: eLife. 2020 Feb 3;9:e48401. doi: 10.7554/eLife.48401 (PMC7064339; doi:10.7554/eLife.48401)
Supplement: Supplementary file 2. [file elife-48401-supp2.docx]

**Supplementary File 2.** Derivation of R_0_

From the main text, we recall that our mean field model takes on the following form:

$\begin{aligned} P \\ \left( | s+P_{A} \right)-\beta P_{S}P_{I}-\mu P_{s}-\rho P_{E}P_{s}-\varepsilon P_{s}+cP_{A} \\ \frac{dP_{s}}{dt}=bP_{D} \end{aligned}$ (1)

$\frac{dP_{A}}{dt}=\rho P_{E}P_{s}+\varepsilon P_{s}-cP_{A}-\mu P_{A}$ (2)

$\frac{dP_{E}}{dt}=\beta P_{S}P_{I}-\sigma P_{E}-\mu P_{E}$ (3)

$\frac{dP_{I}}{dt}=\sigma P_{E}-\alpha P_{I}-\mu P_{I}$ (4)

$\begin{aligned} P \\ \left( | s+P_{A} \right) \\ \frac{dP_{D}}{dt}=\mu\left( P_{s}+P_{E}+P_{I}+P_{A} \right)+\alpha P_{I}-bP_{D} \end{aligned}$ (5)

Parameters are defined according to:

- $b$ = natural birth rate; fixed at .025 to yield stable population sizes in *in vitro* models (Figure 1-figure supplement 5)
- $\mu$ = natural mortality rate; estimated for each cell line by fitting a simple birth-death model to susceptible declines from control trials; values at $\frac{1}{121},$ $\frac{1}{191}$, $\frac{1}{84}$ hours for, respectively, Vero, RoNi/7.1, and PaKiT01 cell lines
- $\beta$ = transmission coefficient; estimated for each cell line/virus/MOI combination by fitting model to data
- $\alpha$ = infection-induced mortality rate (virulence); fixed at $\frac{1}{6}$ hours
- $\sigma$ = incubation rate of cell transitioning from an exposed (E) to an infectious (I) state; measured for each cell line-virus combination as the inverse of the time from initial infection to first observation of infectious virus by GFP under the fluorescent micropscope. Respectively, fixed at the following rates: $\frac{1}{8.38},$ $\frac{1}{7.07}$, $\frac{1}{9.58}$ hours for rVSV-G, rVSV-EBOV, and rVSV-MARV infections on Vero cells; $\frac{1}{8},$ $\frac{1}{6.72}$, $\frac{1}{6.78}$ hours for rVSV-G, rVSV-EBOV, and rVSV-MARV infections on RoNi/7.1 cells; and $\frac{1}{7.17},$ $\frac{1}{6.03}$, $\frac{1}{6.42}$ hours for rVSV-G, rVSV-EBOV, and rVSV-MARV infections on PaKiT01 cells
- $\rho$ = rate of infection-induced acquisition to antiviral state, based on the global proportion of exposed cells in the tissue culture; fixed at 0 under conditions of absent immunity and estimated under conditions of induced and constitutive immunity for all cell line/virus combinations by fitting model to joint data over both MOI-0.001 and 0.0001 data.
- $\varepsilon$ = rate of constitutive acquisition to antiviral state, based on the global proportion of susceptible cells in the tissue culture; fixed at 0 under assumptions of absent and induced immunity; estimated under conditions of constitutive immunity for all cell line/virus combinations by fitting model to joint data over both MOI=0.001 and 0.0001 data.
- $c$ = rate of return to susceptibility from antiviral status; fixed at 0 in all model fits under all assumptions of immunity. Regression from antiviral status to susceptibility occurs on longer time scales than the 200 hour duration of our cell culture trials
- We assume that $P_{s}+P_{A}+P_{E}+P_{I}+P_{D}=1$

We rewrote the equations to model explicit cell densities within a defined well volume. Note that these two systems of equations are equivalent and that parameter values can be used interchangeably across both forms. In the following system, N indicates the total cell capacity of the area modeled:

$\frac{dS}{dt}=bD\frac{\left( S+A \right)}{N}-\frac{\beta SI}{N}-\mu S-\frac{\rho ES}{N}-\varepsilon S+cA$ (6)

$\frac{dA}{dt}=\frac{\rho ES}{N}+\varepsilon S-cA-\mu A$ (7)

$\frac{dE}{dt}=\frac{\beta SI}{N}-\sigma E-\mu E$ (8)

$\frac{dI}{dt}=\sigma E-\alpha I-\mu I$ (9)

$\frac{dD}{dt}=\mu\left( S+E+I+A \right)+\alpha I-bD\frac{\left( S+A \right)}{N}$ (10)

To solve for R_0_, we adopted a next generation matrix approach, after Heffernan, Smith, and Wahl 2005, which we applied to the proportional system of equations (1-5, above). Step one of this process necessitated constructing two transition matrices: the F matrix, which describes how new infections are created and the V matrix which represents host transitions between exposed and infected states.

To build these F and V matrices, we first calculated the value of P_S_ at the disease free equilibrium (DFE, whereby P_I_ and P_E_ are equal to zero. We allow for the presence of antiviral cells (P_A_) at disease free equilibrium, though their equilibrium quantity (and, by consequence, the extent of constitutive immunity at play in the system) depends on certain parameter values. When $\varepsilon=0$, P_A_ will always be 0 at disease free equilibrium. By allowing the extent of constitutive immunity to vary with parameter values, we maintain consistency with the published literature, which suggests that, in constitutively antiviral bat cell lines (i.e. PaKiT01), some interferon-stimulated genes (ISGs) are perpetually expressed, like their IFN-$\alpha$ precursor, while others still require induction upon infection. At DFE, our antiviral population takes on the following form:

$P_{A}=\frac{\varepsilon P_{s}}{\left( c+\mu\right)}$ (11)

And, by extension, our susceptible population can be represented as:

$P_{S}=\frac{\left( b-\mu\right)\left( c+\mu\right)}{b\left( c+\mu+\varepsilon\right)}$ (12)

For cells with either absent or fully induced immunity, for which $\varepsilon=0$, this contracts to:

$P_{S}=\frac{\left( b-\mu\right)}{b}$ (13)

Because we cannot have negative cell proportions at equilibrium, we impose the following constraint on the above parameters:

$\left( b-\mu\right)\left( c+\mu\right)>0$ (14)

When we substitute the above value for P_S_* into the infectious equations, we find:

$\frac{dP_{E}}{dt}=0=\frac{\beta\left( b-\mu\right)\left( c+\mu\right)}{b\left( c+\mu+\varepsilon\right)}P_{I}-\sigma P_{E}-\mu P_{E}$ (15)

$\frac{dP_{I}}{dt}=0=\sigma P_{E}-\alpha P_{I}-\mu P_{I}$ (16)

Our F matrix then takes on the following form:

$F=\left[ \begin{matrix} 0 & \frac{\beta\left( b-\mu\right)\left( c+\mu\right)}{b\left( c+\mu+\varepsilon\right)} \\ 0 & 0 \end{matrix} \right]$ (17)

And the V matrix takes on the following form:

$V=\left[ \begin{matrix} -\left( \sigma+\mu\right) & 0 \\ \sigma& -\left( \alpha+\mu\right) \end{matrix} \right]$ (18)

After Heffernan, Smith, and Wahl 2005, it follows that $G=-FV^{-1}$. Thus, it follows that:

$V^{-1}=\left[ \begin{matrix} \frac{1}{\left( \sigma+\mu\right)} & 0 \\ \frac{\sigma}{\left( \sigma+\mu\right)\left( \alpha+\mu\right)} & \frac{1}{\left( \alpha+\mu\right)} \end{matrix} \right]$ (19)

When this F matrix is multiplied by matrix $-V^{-1}$, we find:

$G=\left[ \begin{matrix} \frac{\beta\sigma\left( b-\mu\right)\left( c+\mu\right)}{b\left( \sigma+\mu\right)\left( \alpha+\mu\right)\left( c+\mu+\varepsilon\right)} & \frac{\beta\left( b-\mu\right)\left( c+\mu\right)}{b\left( \alpha+\mu\right)\left( c+\mu+\varepsilon\right)} \\ 0 & 0 \end{matrix} \right]$ (20)

which, after matrix algebra, yields the following equation for R_0_:

$R_{0}=\frac{\beta\sigma\left( b-\mu\right)\left( c+\mu\right)}{b\left( \sigma+\mu\right)\left( \alpha+\mu\right)\left( c+\mu+\varepsilon\right)}$ (21)

Pathogens can invade a host tissue under conditions of $R_{0}>1$, or when the system satisfies the following inequality:

$\beta\sigma\left( b-\mu\right)\left( c+\mu\right)>b\left( \sigma+\mu\right)\left( \alpha+\mu\right)\left( c+\mu+\varepsilon\right)$ (22)

Finally, we note that for all cells lacking in constitutive antiviral properties ($\varepsilon=0$), R_0_ reduces to:

$R_{0}=\frac{\beta\sigma\left( b-\mu\right)}{b\left( \alpha+\mu\right)\left( \sigma+\mu\right)}$ (23)
