# Supplementary material for: Accelerated viral dynamics in bat cell lines, with implications for zoonotic emergence
Source: eLife. 2020 Feb 3;9:e48401. doi: 10.7554/eLife.48401 (PMC7064339; doi:10.7554/eLife.48401)
Supplement: Supplementary file 3. [file elife-48401-supp3.docx]

**Supplementary File 3.** Special Points from Bifurcation Analysis

| **Special Point** | **ε** | $\boldsymbol{\rho}$ | **β** | **P_I_** |
| --- | --- | --- | --- | --- |
| Branch Point | 0 | .01 | 0.176 | 0 |
| Hopf | 0 | .01 | 1.76 | 0.0133 |
| Branch Point | 0.0001 | .01 | 0.193 | 0 |
| Hopf | 0.0001 | .01 | 2.747 | 0.00944 |
| Branch Point^✝^ | 0.0025 | .01 | 0.615 | 0 |
| Branch Point | 0.0001 | 0 | 0.193 | 0 |
| Hopf | 0.0001 | 0 | 1.407 | 0.0156 |
| Branch Point | 0.0001 | .1 | 0.193 | 0 |
| Hopf | 0.0001 | .1 | 16.918 | 0.00178 |
| Branch Point^✝^ | 0.0001 | 1 | 0.193 | 0 |
| Note: All other parameters in this bifurcation analysis were fixed at the following values: *b* =.025 ; $\mu=0.001$ ; $\sigma=\frac{1}{6}$ ; $\alpha=\frac{1}{6}$ ; $c=0$  ^✝^For these scenarios with high antiviral rates (either induced, $\rho$, or constitutive, ε), no Hopf bifurcation was observed. | | | | |
