# Supplementary material for: Accelerated viral dynamics in bat cell lines, with implications for zoonotic emergence
Source: eLife. 2020 Feb 3;9:e48401. doi: 10.7554/eLife.48401 (PMC7064339; doi:10.7554/eLife.48401)
Supplement: Supplementary file 4. [file elife-48401-supp4.docx]

**Supplementary File 4.** Optimized parameters from all deterministic model outputs and spatial approximations

| **Assumption** | **Cell**  **Line** | **Virus** | **δ AIC^✝^** | **ε**  **[lci – uci] *** | **ρ**  **[lci – uci] *** | **MOI** | **Antiviral Rate** | **β**  **[lci – uci] *** | **Mean Field**  **R_0_** | **Spatial**  **β** | |
| --- | --- | --- | --- | --- | --- | --- | --- | --- | --- | --- | --- |
| Absent | Vero | rVSV-G | 0 | 0 | 0 [0-0] | 0.0001 | 0 | 3.14 [1.86-4.41] | 11.211 | -- | |
|  |  |  |  |  |  | 0.001 | 0 | 2.44 [1.52-3.36] | 8.729 | 24.418 | |
|  |  | rVSV-EBOV | 0 | 0 | 0 [0-0] | 0.0001 | 0 | 1.89 [1.42-2.36] | 6.823 | -- | |
|  |  |  |  |  |  | 0.001 | 0 | 1.5 [1.06-1.94] | 5.416 | 14.996 | |
|  |  | rVSV-MARV | 0 | 0 | 0 [0-0] | 0.0001 | 0 | 1.19 [0.624-1.76] | 4.223 | -- | |
|  |  |  |  |  |  | 0.001 | 0 | 0.975 [0.558-1.39] | 3.454 | 9.752 | |
|  | RoNi/7.1 | rVSV-G | 32.7 | 0 | 0 [0-0] | 0.0001 | 0 | 2.33 [1.39-3.27] | 10.278 | -- | |
|  |  |  |  |  |  | 0.001 | 0 | 2.42 [1.54-3.3] | 10.686 | 24.205 | |
|  |  | rVSV-EBOV | 222 | 0 | 0 [0-0] | 0.0001 | 0 | 0.609 [.367-0.851] | 2.707 | -- | |
|  |  |  |  |  |  | 0.001 | 0 | 0.675 [.466-0.885] | 3.001 | 6.753 | |
|  |  | rVSV-MARV | 3.36 | 0 | 0 [0-0] | 0.0001 | 0 | 1.22 [0.891-1.54] | 5.405 | -- | |
|  |  |  |  |  |  | 0.001 | 0 | 1.23 [0.924-1.53] | 5.457 | 12.284 | |
|  | PaKiT01 | rVSV-G | 175 | 0 | 0 [0-0] | 0.0001 | 0 | 2.48 [1.38-3.58] | 6.689 | -- | |
|  |  |  |  |  |  | 0.001 | 0 | 2.56 [1.58-3.55] | 6.917 | 25.639 | |
|  |  | rVSV-EBOV | 27.9 | 0 | 0 [0-0] | 0.0001 | 0 | 0.663 [.399-0.927] | 1.811 | -- | |
|  |  |  |  |  |  | 0.001 | 0 | 0.837 [0.215-1.46] | 2.287 | 8.37 | |
|  |  | rVSV-MARV | 665 | 0 | 0 [0-0] | 0.0001 | 0 | 0.393 [0-2.2] | 1.068 | -- | |
|  |  |  |  |  |  | 0.001 | 0 | 0.379 [0-1.66] | 1.03 | 3.785 | |
| Induced | Vero | rVSV-G | 2 | 0 [0-0] | 4.55x10^-10^  [0-3.92x10^-7^] | 0.0001 | 3.04x10^-14^ | 3.14 [1.86-4.41] | 11.211 | -- | |
|  |  |  |  |  |  | 0.001 | 3.04x10^-13^ | 2.44 [1.52-3.36] | 8.727 | 24.413 | |
|  |  | rVSV-EBOV | 2 | 0 [0-0] | 4.64x10^-8^  [0-4.38x10^-7^] | 0.0001 | 3.11x10^-12^ | 1.89 [1.42-2.36] | 6.824 | -- | |
|  |  |  |  |  |  | 0.001 | 3.10x10^-11^ | 1.5 [1.06-1.94] | 5.416 | 14.996 | |
|  |  | rVSV-MARV | 2 | 0 [0-0] | 2.05x10^-7^  [0-5.97x10^-7^] | 0.0001 | 1.37x10^-11^ | 1.19 [0.624-1.76] | 4.223 | -- | |
|  |  |  |  |  |  | 0.001 | 1.37x10^-10^ | 0.975 [0.558-1.39] | 3.454 | 9.752 | |
|  | RoNi/7.1 | rVSV-G | 0 | 0 [0-0] | 0.089 [0-0.432] | 0.0001 | 7.03x10^-6^ | 2.38 [1.37-3.39] | 10.504 | -- | |
|  |  |  |  |  |  | 0.001 | 7.03x10^-5^ | 2.47 [1.49-3.45] | 10.907 | 24.705 | |
|  |  | rVSV-EBOV | 0 | 0 [0-0] | 0.0363  [0-0.343] | 0.0001 | 2.87x10^-6^ | .622 [.336-.907] | 2.763 | -- | |
|  |  |  |  |  |  | 0.001 | 2.87x10^-5^ | .685 [.451-.919] | 3.043 | 6.849 | |
|  |  | rVSV-MARV | 0 | 0 [0-0] | 0.0177  [0-0.257] | 0.0001 | 1.40x10^-6^ | 1.22 [.882-1.56] | 5.424 | -- | |
|  |  |  |  |  |  | 0.001 | 1.40x10^-5^ | 1.23 [.917-1.55] | 5.475 | 12.324 | |
|  | PaKiT01 | rVSV-G | 29.9 | 0 [0-0] | 0.311  [0-1.02] | 0.0001 | 1.63x10^-5^ | 2.8 [1.43-4.17] | 7.562 | -- | |
|  |  |  |  |  |  | 0.001 | 0.000162 | 2.72 [1.4-4.03] | 7.334 | 27.183 | |
|  |  | rVSV-EBOV | 1020 | 0 [0-0] | 0.881  [0-3.32] | 0.0001 | 0.0000461 | 1.08 [0-2.65] | 2.947 | -- | |
|  |  |  |  |  |  | 0.001 | 0.000461 | 1.55 [0-4.08] | 4.233 | 15.495 | |
|  |  | rVSV-MARV | 0 | 0 [0-0] | 13.1  [0-37.9] | 0.0001 | 0.000687 | 0.698 [0-10.1] | 1.9 | -- | |
|  |  |  |  |  |  | 0.001 | 0.00687 | 3.25 [0-41.3] | 8.828 | 32.452 | |
| Constitutive | Vero | rVSV-G | 4 | 2.86x10^-24^  [0-0.00237] | 3.05x10^-8^  [0-0.00237] | 0.0001 | 2.04x10^-12^ | 3.14 [1.81-4.47] | 11.211 | -- | |
|  |  |  |  |  |  | 0.001 | 2.04x10^-11^ | 2.44 [1.49-3.4] | 8.73 | 24.42 | |
|  |  | rVSV-EBOV | 4 | 5.42x10^-20^  [0-0.00202] | 2.39x10^-8^  [0-0.00202] | 0.0001 | 1.60x10^-12^ | 1.89 [1.39-2.39] | 6.824 | -- | |
|  |  |  |  |  |  | 0.001 | 1.60x10^-11^ | 1.5 [1.03-1.97] | 5.416 | 14.996 | |
|  |  | rVSV-MARV | 247 | 0.00725  [0.00325-0.0113] | 4.84x10^-10^  [0-3.92x10^-7^] | 0.0001 | 0.00259 | 2.42 [0.672-4.16] | 4.565 | -- | |
|  |  |  |  |  |  | 0.001 | 0.00259 | 1.83 [0.626-3.04] | 3.464 | 18.347 | |
|  | RoNi/7.1 | rVSV-G | 2 | 6.14x10^-10^  [0-3.93x10^-7^] | 0.089  [0-0.432] | 0.0001 | 7.03x10^-6^ | 2.38 [1.37-3.39] | 10.504 | -- | |
|  |  |  |  |  |  | 0.001 | 7.03x10^-5^ | 2.47 [1.49-3.45] | 10.906 | 24.703 | |
|  |  | rVSV-EBOV | 2.01 | 1.08x10^-9^  [0-3.93x10^-7^] | 0.0366  [0-0.346] | 0.0001 | 2.90x10^-6^ | 0.622 [0.336-0.908] | 2.763 | -- | |
|  |  |  |  |  |  | 0.001 | 2.89x10^-5^ | 0.685 [0.45-0.919] | 3.042 | 6.846 | |
|  |  | rVSV-MARV | 2 | 5.54x10^-10^  [0-3.93x10^-7^] | 0.0176  [0-0.257] | 0.0001 | 1.40x10^-6^ | 1.22 [0.882-1.56] | 5.424 | -- | |
|  |  |  |  |  |  | 0.001 | 1.39x10^-5^ | 1.23 [0.917-1.55] | 5.475 | 12.324 | |
|  | PaKiT01 | rVSV-G | 0 | 0.00602  [0-0.019] | 8.26x10^-8^  [0-4.75x10^-7^] | 0.0001 | 0.00209 | 3.68 [0.919-6.44] | 6.593 | -- | |
|  |  |  |  |  |  | 0.001 | 0.00209 | 3.45 [1.07-5.84] | 6.189 | 34.516 | |
|  |  | rVSV-EBOV | 0 | 0.0478  [0-0.0958] | 4.46x10^-8^  [0-4.37x10^-7^] | 0.0001 | 0.00499 | 15.6 [12.7-18.5] | 8.518 | -- | |
|  |  |  |  |  |  | 0.001 | 0.00499 | 34.5 [28.7-40.2] | 18.823 | 344.821 | |
|  |  | rVSV-MARV | 2 | 3.99x10^-7^  [6.86x10^-9^-7.91x10^-7^] | 13.1  [1.48-24.8] | 0.0001 | 0.000687 | 0.699 [0-10.1] | 1.902 | -- | |
|  |  |  |  |  |  | 0.001 | 0.00687 | 3.24 [0-14.5] | 8.815 | 32.406 | |
| ^✝^Best fit models indicated at δ-AIC = 0 are presented in Table 1 and Figure 1 and 3 (main text)  * lci = lower and uci = upper 95% confidence interval. No confidence interval is shown for spatial β which was fixed at 10 times the estimated mean for the mean field model fits when paired with equivalent values of ε and ρ.  All other parameters were fixed at the following values: b=.025 (mean field), .15 (spatial); α = 1/6; c=0; μ= 1/121 (Vero), 1/191(RoNi/7.1, and 1/84 (PaKiT01) | | | | | | | | | | |  |
