# Supplementary material for: Accelerated viral dynamics in bat cell lines, with implications for zoonotic emergence
Source: eLife. 2020 Feb 3;9:e48401. doi: 10.7554/eLife.48401 (PMC7064339; doi:10.7554/eLife.48401)
Supplement: Supplementary file 5. [file elife-48401-supp5.docx]

**Supplementary File 5.** Justification for parameter increase from mean field to spatial model.

Because spatial configurations elevate thresholds for pathogen invasion, as well for host cell persistence (Webb, Keeling, and Boots 2007), we were forced to elevate both birth rates (*b*) and transmission rates ($\beta$) above those used in the mean field model to apply to the spatial context. We here justify our chosen values for parameter increase:

In Webb, Keeling, and Boots 2007, the authors approximate spatial dynamics using a series of differential equations tracking the pairwise neighbor-neighbor interactions of a ﻿regular square lattice with a Von Neumann neighborhood. Webb *et al.* represent local reproduction as $b(1-L_{b})/z$ and local transmission as $\beta(1-L_{I})/z$ where z is the number of near-neighbor pairs, $L_{b}$ is the proportion of global reproduction (births), and $L_{I}$is the proportion of global transmission in the system. Note that we altered the reproduction parameter *r* to *b* to reflect our own nomenclature.

Cumulatively, we can represent total reproduction ($b$) and total transmission ($\beta_{tot}$) in a system with both local and global effects as:

$b_{tot}=\frac{b(1-L_{b})}{z}+ bL_{b}$ (1)

$\beta_{tot}=\frac{\beta(1-L_{I})}{z}+ \beta L_{I}$ (2)

Our goal is to find a scalar ($q$) by which to multiply the mean field birth ($q_{b}$) and transmission ($q_{\beta}$) rates to values appropriate for the spatial context, such that:

$b_{spatial}=q_{b}b_{mean}$ (3)

$\beta_{spatial}=q_{\beta}\beta_{mean}$ (4)

Since we know that $L_{b}$ and $L_{I}$ both equal 1 in the mean field model, we can represent these interactions as:

$b_{mean}=\frac{{q_{b}b}_{mean}(1-L_{b})}{z}+ {{q_{b}b}_{mean}L}_{b}$ (5)

$\beta_{mean}=\frac{{q_{\beta}\beta}_{mean}(1-L_{b})}{z}+ {{q_{\beta}\beta}_{mean}L}_{b}$ (6)

And then solve for ($q_{b}$) and ($q_{\beta}$):

$q_{b}=\frac{z}{zL_{b}+1-L_{b}}$ (7)

$q_{\beta}=\frac{z}{zL_{I}+1-L_{I}}$ (8)

It is easy to see how, in the perfect pair approximation, when $L_{b}$or $L_{I}=0$, $q=$ z meaning that the mean field transmission rate is simply multiplied by the number of nearest neighbors.

Equally, in the mean field context, when $L_{b}$or $L_{I}=1$, $q=$ 1.

From equation (7), it is straightforward to calculate ($q_{b}$) for our spatial model, since births are permitted only at the nearest-neighbor interface, and each cell has six adjacent neighbors. As such:

$q_{b}=\frac{6}{6*0+1-0}=6$ (9)

To this end, we multiplied all mean field birth rates (b=0.025) by 6 to equal .15 in the spatial model.

The dynamics are slightly more complicated in the case of the transmission rate modifier ($q_{\beta}$) because our spatial model allows cells to influence infection up to three ‘rings’ out from the nearest neighbor, such that each cell is affected by the proportion infectious in a 36-cell vicinity. These dynamics accurately reflect viral transmission, which, even in a plaque assay, can diffuse beyond the immediate neighbor-neighbor boundary, especially in a ten-minute timestep.

As such, we chose to represent transmission processes in the spatial model under assumptions of $L_{I}=.075$, allowing ~7.5% of transmission to be modeled globally and leading to a less extreme multiplication of the mean field transmission rate than assumed under the perfect pair approximation ($q=$ z) illustrated in equation (9) above. Under these new assumptions, we modify the mean field transmission rate for the spatial context as follows:

$q_{\beta}=\frac{36}{36*.075+1-.075}=9.93$ (10)

Since this is an approximation and all transitions occur stochastically, we round $q_{\beta}$ up to multiply all mean field transmission rates by ten in the spatial context.
