# Supplementary material for: Accelerated viral dynamics in bat cell lines, with implications for zoonotic emergence
Source: eLife. 2020 Feb 3;9:e48401. doi: 10.7554/eLife.48401 (PMC7064339; doi:10.7554/eLife.48401)
Supplement: Supplementary file 6. [file elife-48401-supp6.docx]

**Supplementary File 6.** Primers for qPCR

| **Species** | **Cell Line** | **Gene** | **Primer** | **Sequence** | **Original Publication** |  |
| --- | --- | --- | --- | --- | --- | --- |
| ***Rousettus aegyptiacus*** | RoNi/7.1 | β-Actin | Fwd | GGCTCCCAGCACAATGAAGA | Biesold *et al.*, 2011 | |
|  |  |  | Rev | GGAGCCGCCGATCCA | Biesold *et al.*, 2011 | |
|  |  | IFN-α | Fwd | GAGACTCCCCTGCTGGATGA | Cowled *et al.*, 2011 | |
|  |  |  | Rev | ATAGAGGGTGATTCTCTGGAAGTATTTC | Cowled *et al.*, 2011 | |
|  |  | IFN-β | Fwd | CAGCTATTTCCATGAGCTACAACTTG | Biesold *et al.*, 2011 | |
|  |  |  | Rev | TTAACTGCCACAGGAGCTTCAG | Biesold *et al.*, 2011 | |
| *Pteropus alecto* | PaKiT01 | β-Actin | Fwd | GGCTCCCAGCACAATGAAGA | Biesold *et al.*, 2011 | |
|  |  |  | Rev | GGAGCCGCCGATCCA | Biesold *et al.*, 2011 | |
|  |  | IFN-α | Fwd | GAGACTCCCCTGCTGGATGA | Cowled *et al.*, 2011 | |
|  |  |  | Rev | ATAGAGGGTGATTCTCTGGAAGTATTTC | Cowled *et al.*, 2011 | |
|  |  | IFN-β | Fwd | CTCTAGCACTGGCTGGAATGAA | Cowled *et al.*, 2011 | |
|  |  |  | Rev | TGCCCACCGAGTGTCTCA | Cowled *et al.*, 2011 | |
