# Supplementary material for: Accelerated viral dynamics in bat cell lines, with implications for zoonotic emergence
Source: eLife. 2020 Feb 3;9:e48401. doi: 10.7554/eLife.48401 (PMC7064339; doi:10.7554/eLife.48401)
Supplement: Supplementary file 7. [file elife-48401-supp7.docx]

**Supplementary File 7.** Detailed methods for image and image data processing.

*Image Processing.*

All image processing and data analysis was carried out in R version 3.6 for MacIntosh (R Core Team 2019). Original images were imported into R and processed via the package EBImage (Pau et al. 2010). Composite images of each well were first split into the 36 or 64-subframes from which they were composed (each subframe represents the visual region of focus for the microscope at the time of imaging). Each subframe was trimmed (to remove border effects), processed individually, and recompiled post-processing into binary form, such that light-colored regions of the original image were assigned a value of 1 (white), and dark regions were assigned a value of 0 (black). In the case of images of GFP-expressing cells, these white regions corresponded to “infectious” cells, while in images of Hoechst-stained nuclei, they indicated live, “uninfected” cells.

Microscope focus was poor for control wells and for subframes early in the time series of each trial before GFP expression became apparent, and the original versions of any such subframes were light gray and grainy. Our image processing code identified these subframes as any which possessed a mean pixel value greater than .25 (a value substantially higher than any subframes in which true GFP-expressing or Hoechst-stained cells were visible) and subsequently converted the entire frame to 0 (black).

All other subframes were processed following thresholding methods that have been previously described by the authors of EBImage (Pau et al. 2010). As a first pass, all pixels excepting the top 25% lightest pixels tallied were converted to 0 (black). Next, each image frame was walked through a series of secondary thresholding steps using if-else statements in R, such that the lightness threshold for "infectious" status was elevated in frames which were lighter overall due to manual variation in imaging and focusing. Processed subframes were then reconstructed into composite binary images, which were manually checked against original images to ensure consistent and reliable results.

Post-processing into binary form, the number of discrete shapes with value of 1 were tabulated within each image, using the max(bwlabel(X)) function in EBimage, to determine a cell count per image, again corresponding to a count of infectious cells for GFP-expressing images and to a count of uninfected cells for Hoechst stain images. All image processing and counting scripts, in addition to the resulting data, are freely available for download at the following FigShare repository: DOI: 10.6084/m9.figshare.8312807.

*Image Data processing.*

GFP-expressing images were processed and cells counted across the duration of each infection trial, thus generating a time series of infectious cells. For a subset of plates, infection was terminated, and cells were fixed, Hoechst stained, and imaged at periodic intervals across the duration of the time series. Processing of these images thus allowed for construction of a corresponding time series of live, uninfected cells. Because of logistical constraints (i.e. many plates of simultaneously running infection trials and only one available imaging microscope), the time course of imaging across the duration of each trial was quite variable. As such, we fitted a series of statistical models to our raw image data to reconstruct reliable values of the infectious proportion of each well per hour for each distinct trial in all cell line–virus-MOI combinations (Figure 1-figure supplement 2-3).

There was considerable natural variation in initial cell counts across each trial, resulting from subtle differences in the seeding density and growth duration of time until the trial was initiated (when wells were subjectively deemed to have reached “90% confluency”). Baseline cell counts were also different across our three cell lines, which varied substantially in natural size. To correct for this variation, we opted to model the proportion of infectious cell spaces per hour for each well, rather than rely on the raw count data. To this end, we collected the maximum number of live cells counted in susceptible control wells at timepoint 0 and set this count to a rough measure of 100% well occupancy for the cell line in question. Using this method, maximum cell counts were, respectively, 103712, 82308, and 92233 for Vero, RoNi/7.1, and PaKiT01 cell lines, reflecting innate variation in cell sizes. We then converted all cell counts tabulated via our image processing code across the infectious time trials into proportions by dividing the counts by the total number of possible cell spaces for the cell line in question. Though clearly subject to some error, these methods nonetheless maintained internal consistency in our counting methods and generated reasonable time series. We originally experimented with directly tabulating the proportion of infected versus uninfected space in our binary images; however, this approach impaired our ability to generalize across more or less densely seeded trials, as well as trials on cell lines of disparate sizes. As such, we adopted the count-to-proportion methods described here.

To generate an infectious time series of evenly distributed time steps against which to fit our mean field mechanistic model, we next fit a series of statistical models to the proportional data produced from the image processing methods described above. For the GFP-expressing data, we used the mgcv package in R (Wood 2001) to fit generalized additive models (GAMs) in the Gaussian family, with time elapsed (in hours) post infection as a predictor variable for proportion of infectious cells (the response variable). We fit a separate GAM model to each unique cell – virus – MOI combination, incorporating a random effect of well ID (such that each trial was modeled individually), and we fixed the smoothing parameter at k=7 for all trials, as recommended by the package author (Wood 2001). The gam.predict() function was used to return an estimate of infectious proportions per hour across the duration of each time series for each cell-virus-MOI combination.

The uninfected counts from the Hoechst stain data were much less numerous since each count required termination of the infection trial and fixation of cells; by definition, only one data point could be produced per trial. Due to this relative scarcity, we opted to fit a more standard linear regression model, again in the Gaussian family, to these data, rather than using the data-hungry GAM methods employed above. As before, we set time elapsed post infection as the predictor for the Hoechst stain data and produced a unique estimate of the proportion of uninfected cells per hour across the duration of the longest-observed trial. No random effects were included in this model, and the resulting time series were used to estimate natural mortality rates for each cell line, when fit to control well data depicting natural susceptible decline (Figure 1-figure supplement 7).
